# Running Velocity and Longitudinal Bending Stiffness Influence the Asymmetry of Kinematic Variables of the Lower Limb Joints

**DOI:** 10.3390/bioengineering9110607

**Published:** 2022-10-23

**Authors:** Qian Liu, Hairong Chen, Yang Song, Nykytiuk Alla, Gusztáv Fekete, Jianpeng Li, Yaodong Gu

**Affiliations:** 1 Faculty of Sports Science, Ningbo University, Ningbo 315211, China; 2 Faculty of Engineering, University of Szeged, 6720 Szeged, Hungary; 3 Savaria Institute of Technology, Faculty of Informatics, Eötvös Loránd University, 9700 Szombathely, Hungary

**Keywords:** longitudinal bending stiffness, running velocity, kinematic, asymmetry, lower limb

## Abstract

Running-related limb asymmetries suggest specific sports injuries and recovery circumstances. It is debatable if running speed affected asymmetry, and more research is required to determine how longitudinal bending stiffness (LBS) affected asymmetry. The purpose of this study was to investigate the influence of running velocity and LBS on kinematic characteristics of the hip, knee, ankle, metatarsophalangeal joint (MTP) and the corresponding asymmetry. Kinematic (200 Hz) running stance phase data were collected bilaterally for 16 healthy male recreational runners (age: 23.13 ± 1.17, height: 175.2 ± 1.6 cm, body mass: 75.7 ± 3.6 kg, BMI: 24.7 ± 1.3 kg/m^2^) running on a force plate at three different velocities (10, 12 and 14 km/h) and three increasing-LBS shoes in a randomized order. The symmetry angle (SA) was calculated to quantify gait asymmetry magnitude at each running velocity and LBS. Changes in running velocity and LBS led to differences in kinematic variables between the hip, knee, ankle and MTP (*p* < 0.05). Significant changes in SA caused by running velocity were found in the knee flexion angle (*p* = 0.001) and flexion angle peak velocity (*p* < 0.001), ankle plantarflexion angle (*p* = 0.001) and plantarflexion angle peak velocity (*p* = 0.043) and MTP dorsiflexion angle (*p* = 0.001) and dorsiflexion angle peak velocity (*p* = 0.019). A significant change in the SA caused by LBS was found in the MTP dorsiflexion peak angle velocity (*p* = 0.014). There were interaction effects between running velocity and LBS on the MTP plantarflexion angle (*p* = 0.033) and plantarflexion angle peak velocity (*p* = 0.038). These findings indicate the existence of bilateral lower limb asymmetry. Meanwhile, it was proved that running velocity and LBS can influence the asymmetry of lower limb joints. Additionally, there was an interaction between running velocity and LBS on the asymmetry of the lower limb. These findings can provide some information for sports injuries, such as metatarsal stress fractures and anterior cruciate ligament injuries. They can also provide some useful information for running velocities and running shoes.

## 1. Introduction

Longitudinal bending stiffness (LBS) is a footwear property that is known as one key consideration for footwear performance development [1,2]. A suitable LBS is also thought to be a crucial element in comfort and athletic performance [2,3]. The fast development of footwear technology has increased awareness of LBS. The majority of studies have also found a link between LBS and sports injuries [1,4,5]. Increasing the forefoot bending stiffness of footwear may help with injury prevention in particular by preventing excessive forefoot extension during sports activities to reduce the incidence of injuries such as turf toe [4]. Studies suggest that the stiffness of the forefoot bending may have an effect on the prevalence of metatarsal stress fractures. Stiff shoes cause the center of pressure under the foot to shift more anteriorly and alter the peak pressures acting on various foot regions [1]. Performance in long-distance running is influenced by running economy (RE) [6]. In a study by Roy and Stefanyshyn et al. [7], approximately 1% metabolic energy savings were observed when participants ran in a stiff midsole. In addition, studies had shown that stiff carbon-fiber plates may reduce the energetic cost of running by 4% [8]. These suggested that enhancing the midsole’s LBS would enhance RE.

The bulk of research overlooked the asymmetry between the bilateral lower limbs and instead used the unilateral dominant leg to represent the overall performance of the bilateral lower limbs [9] in order to simplify data collection and processing. The human body’s symmetry, however, is not perfect. There are asymmetries between the dominant and non-dominant legs, according to studies [10]. Lower limb asymmetry was not only caused by genetics and hormones, but also by biomechanical variables [11,12]. The differences in the load-related dynamics of the bilateral limbs may lead to asymmetry [12]. The existence of lower limb asymmetry was demonstrated in an experiment that included jumping–landing exercises. The knee moment of the dominant leg was greater than that of the non-dominant limb [13]. Additionally, it has been shown that knee flexion moment asymmetry predicts re-injury in athletes who had an anterior cruciate ligament reconstruction [14]. In earlier studies, the symmetry angle (SA), which reflects the symmetry of kinematic and kinetic variables of lower extremity joints, was frequently used [15]. In comparison to pre-fatigue, the SA of the knee flexion angle, hip flexion angle and hip extension angle was significantly higher in post-fatigue. In other words, after running, the joint asymmetry of the lower extremities becomes worse [16].

The biomechanical asymmetry of the lower limb can provide some information related to sports injuries. Idiopathic scoliosis may be caused by pelvic and hip angle asymmetry [17]. Kotwicki et al. [18], in a comparison of hip range of motion (ROM) asymmetry between scoliosis and normal adolescents, found that the scoliosis group showed greater asymmetry. For evaluating athletes’ return to the field following anterior cruciate ligament surgery and recuperation, the asymmetry of knee ROM provides important reference data [19]. Although asymmetry has often been often considered a manifestation of pathology, for some lower limb joint movements, the asymmetry in the range of variation of bilateral lower limbs in healthy people remains to be investigated [20].

Lower limb asymmetry has been shown to increase with walking velocity, suggesting that running may result in even more asymmetry. Biomechanical asymmetry is not detrimental during walking tasks; however, greater biomechanical demands are imposed on the musculoskeletal system during running [9]. Mo et al. discovered that among recreational runners, SA altered nonlinearly and displayed an approximately U-shaped trend across velocities [21]. Running velocity, in contrast, had no impact on the asymmetry of the kinematic characteristics of the joints in the lower limbs, according to Jiang et al. [11]. The negative work performed by the metatarsophalangeal joint (MTP) decreased with the increase in LBS and significantly changed the mechanical properties of the ankle and knee [22]. The MTP dorsiflexion angle and dorsiflexion angle velocity reduced with an increase in LBS to lessen the chance of forefoot injury [1,23]. Research on the kinematic variations of lower limb joints regarding LBS is very extensive. Research on the impact of LBS on lower limb asymmetry is lacking, however. Additionally, the impact of running speed on the kinematics of the lower limbs has always been debatable.

As a result, the purpose of this study was to explore the difference and asymmetry in lower limb kinematic variables when wearing increasing-LBS shoes at different running velocities. We analyzed the influence of running velocity and LBS on angles, angle peak velocities and the SA of the hip, knee, ankle and MTP.

## 2. Materials and Methods

### 2.1. Participants

The sample size was calculated using G*Power 3.1 (Franz Faul, Germany) for univariate analysis of variance for detecting a medium Cohen’s effect size (d = 0.4), α error probability = 0.05 and power (1 − β) = 0.95. Based on these parameters, it was estimated that a minimum of 14 participants would be required for this study [24]. A total of 16 healthy males (age: 23.13 ± 1.17, height: 175.2 ± 1.6 cm, body mass: 75.7 ± 3.6 kg, BMI: 24.7 ± 1.3 kg/m^2^) who were recreational runners (no formal running competition, training at least 3 times a week) were recruited [11,25]. The recruitment criteria for recreational runners in this experiment were running for at least 6 months and running a minimum distance of 10 km per week and having the right-side limb as the dominant limb [26]. The dominant limb was defined as the preferred leg when kicking a ball. All participants were free from health problems and/or neuromuscular disorders and/or known gait impairments, and had had no lower limb injuries in the previous six months. All participants were rearfoot strikers and were recruited from Ningbo University for this study. Before the experiment, all participants gave written consent. The study was approved by the Ethics Committee of the Research Institute at Ningbo University.

### 2.2. The Experimental Process

**Figure 1:**
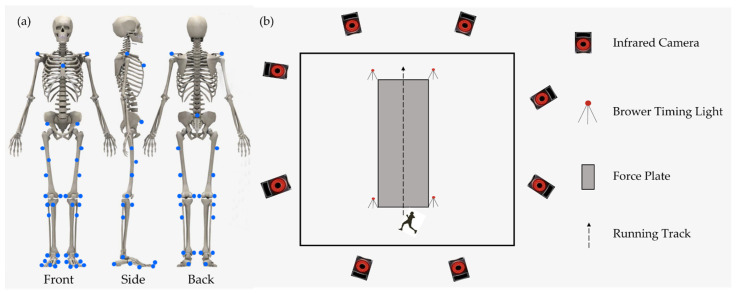
(**a**) The front, side and back positions of markers. Blue dots: markers. (**b**) Illustration of experiment design for collecting the kinematics data during the running stance phase.

The general process of the experiment is shown in Figure 1b. Before the formal test, participants had 10 min to warm up and familiarize themselves with experimental settings. In the first step of the formal test, each participant was asked to stand on a force platform to collect static coordinates by standing parallel to the *Y*-axis of the force platform with arms crossed over shoulders and eyes looking forward until the full static coordinates were captured. All participants were allowed three trials to familiarize themselves with the test maneuvers before the formal test. During the test, participants were asked to wear Shoe 1 (S1), Shoe 2 (S2) and Shoe 3 (S3) at 10 ± 5% km/h (V1), 12 ± 5% km/h (V2) and 14 ± 5% km/h (V3), respectively, over a 10 m track [5,11,15,27]. The shoe information is shown in Table 1. Additionally, participants were asked to complete a full gait cycle on a 2 m force platform (Kistler, Winterthur, Switzerland) located in the middle of the track. Five trials were achieved to gather eligible data on the dominant leg, in which the running speed of the participant had less than 5% variance and was within 5% of the predefined running speed. The full gait cycle was defined as the time from the right heel strike to the left forefoot coming off the ground in this test. The LBS values of the shoes were measured by a rotational axis material-testing machine (Instron ElectroPuls E1000, Norwood, MA, USA). The force platform recorded the ground reaction force at 1000 Hz to distinguish a complete gait cycle. An eight-camera motion capture system (Vicon Metrics Ltd., Oxford, United Kingdom) was used to record running kinematic data during the stance phase at a frequency of 200 Hz. A threshold of 20 N on the vertical ground reaction force was applied to identify the initial foot contact and toe-off. To manage running velocity, Brower timing lights (Brower Timing System, Draper, UT, USA) were used. Before the experiment, participants were asked to apply 38 reflective markers (diameter: 14 mm) on their bodies. The specific positions of the markers are shown in Figure 1a.

### 2.3. Data Analysis

This study focused on the sagittal planes of the hip, knee, ankle and MTP. Dominant variation in the sagittal plane is reported to occur during running [28]. Marker trajectories were filtered by zero-latency fourth-order Butterworth low-pass filters at 12 Hz. The C3D file data were converted to formats recognized in OpenSim 4.3 (.mot and .trc) by Matlab R2016a (The MathWorks, Natick, MA, USA), and then imported into OpenSim for data processing [29]. A musculoskeletal model in OpenSim (gait 2392) was used. The model was scaled using the participant’s marker point location and weight in a static calibration. The static weight of each marker was manually adjusted according to the root mean square (RMS) error value (less than 0.02) between the experimental and virtual markers in the model until it was adjusted to the appropriate position before applying the scaled model to the data calculation. The joint angles were calculated using the inverse kinematics (IK) calculation tool in OpenSim, and the results were optimized using least squares to minimize the error between the experimental and virtual markers.

SA was used to evaluate the biomechanical symmetry of the participants’ dominant limb and non-dominant limb. SA can be calculated as follows:

$$\mathrm{SA}=\frac{\left(45^{{}^{\circ}}-\arctan\left(X_{left}/X_{right}\right)\right)}{90{}^{\circ}}\times 100\%$$

If

$$\left(45^{{}^{\circ}}-\arctan\left(X_{left}/X_{right}\right)\right)$$

Then

$$\mathrm{SA}=\frac{\left(45^{{}^{\circ}}-\arctan\left(X_{left}/X_{right}\right)-180^{{}^{\circ}}\right)}{90{}^{\circ}}\times 100\%$$

*X_left_* represents the kinematic variables of the left lower limb, and *X_right_* represents the kinematic variables of the right lower limb. A score of 0% suggests perfect symmetry and 100% suggests perfect asymmetry between the right and left leg [30].

### 2.4. Statistical Analysis

SPSS 26.0 (SPSS, Chicago, IL, USA) software was used for statistical analysis.

Descriptive statistics were provided as means and standard deviations (SDs). Tests for normality and homogeneity of variances (Shapiro–Wilk and Levene’s, respectively) were conducted on all SA data before the analysis. Univariate ANOVA was performed to determine the effects of running velocity and LBS on bilateral joint angle and angle peak velocity asymmetry during running. In the presence of interaction, simple effect comparisons were performed. Pairwise comparisons with Bonferroni were used post hoc to further analyze significant effects of running velocity, LBS and interaction. Effect sizes were calculated using partial eta^2^ (*η*^2^), with the relative magnitude of any differences expressed as a standard criterion: small effect size (0.01 < *η*^2^ < 0.05), medium effect size (0.06 < *η*^2^ < 0.14) and large effect size (*η*^2^ > 0.14) [31,32,33]. Paired *t*-tests assessed differences in joint angles and angle peak velocities between legs at different running velocities and in increasing-LBS shoes. This was primarily used to investigate if running at different velocities while wearing increasing-LBS shoes caused variations in the kinematic characteristics of bilateral lower limbs. The level of statistical significance was set at *p* < 0.05.

Joint angles and joint angle velocities during the running stance phase were compared using one-dimension statistical parameter mapping (SPM1d). For SPM1d, kinematic data for each step were time-normalized to the stance phase (101 data points per stance phase). Biomechanical differences between the right and left lower limb of each running velocity and LBS were obtained by statistically examining the entire time series using SPM1d with post hoc paired *t*-tests. All SPM1d analyses were conducted in MATLAB R2016a (The MathWorks, MA, USA) using the open-source software package spm1d 8 [34] (www.spm1d.org, accessed on 2 May 2022).

## 3. Results

**Figure 2:**
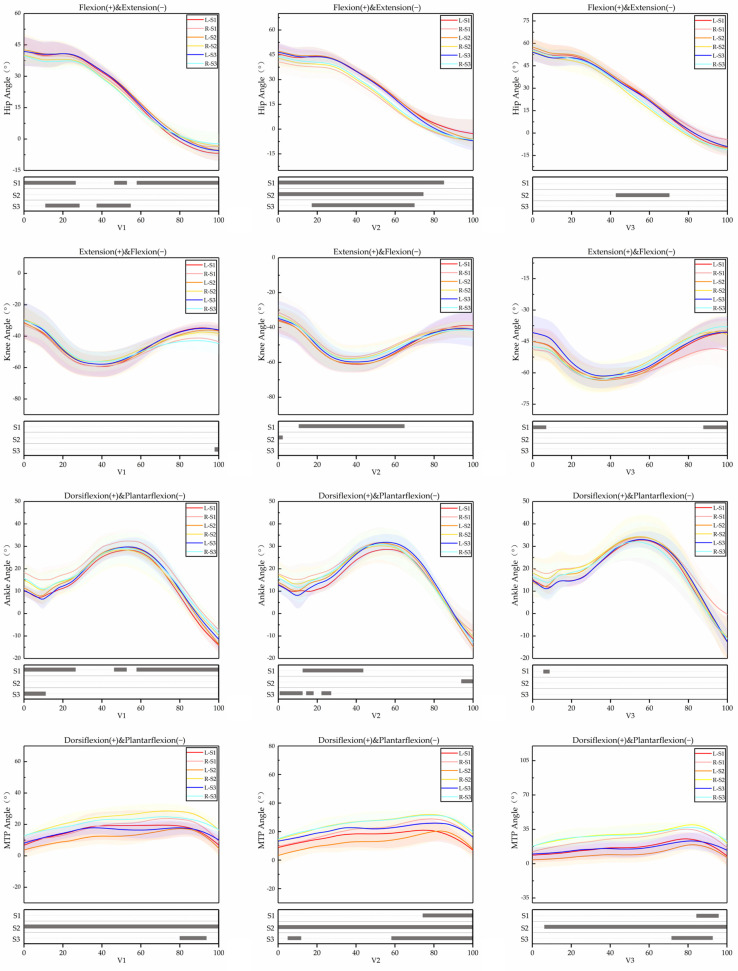
Descriptive results of angles between left lower limb and right lower limb SPM during running at V1, V2 and V3. L = left lower limb, R = right lower limb.

**Figure 3:**
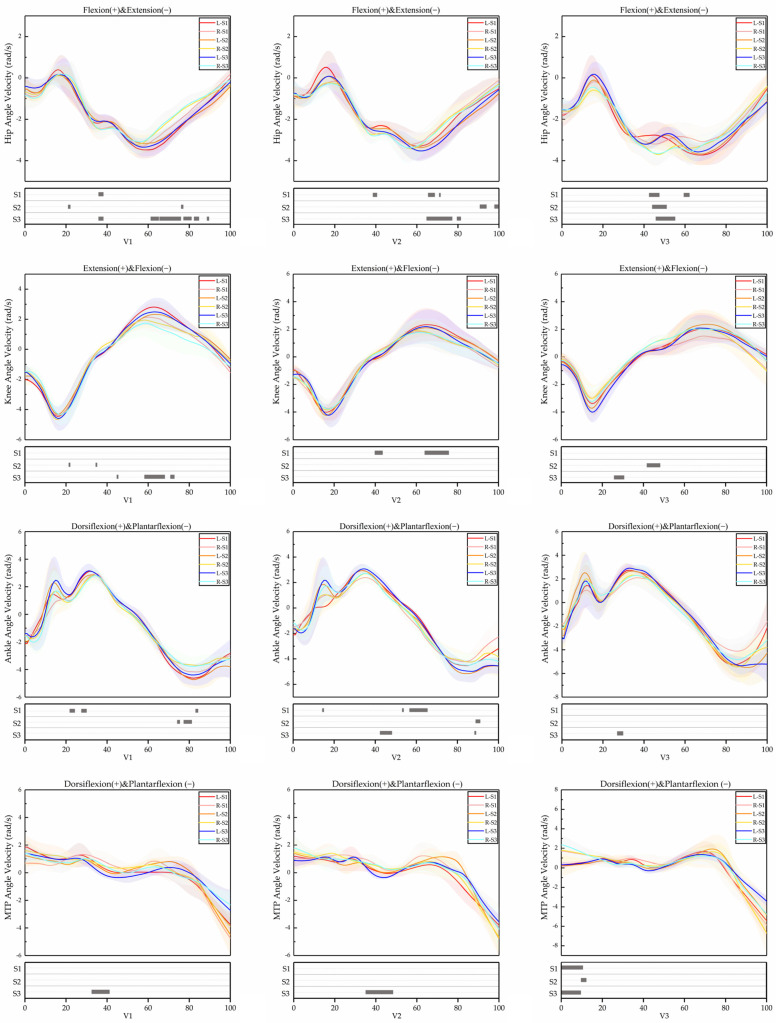
Descriptive results of angle velocities between left lower limb and right lower limb SPM during running at V1, V2 and V3. L = left lower limb, R = right lower limb.

The effects of running velocity and LBS on kinematic variables of lower limb joints and SA were shown in Table 2 and Table 3, respectively. Figure 2 and Figure 3 show the angle and angle velocity changes in the hip, knee, ankle and MTP.

### 3.1. The Influence of Running Velocity and LBS on Lower Limb Joints’ Kinematic Variables

#### 3.1.1. Hip

There were significant differences in the hip extension angle when the participants ran at V1 wearing S1, S2 and S3 (*p* < 0.05). The extension angle and extension angle peak velocity were significantly different when they ran at V2 wearing S3 (*p* < 0.05).

#### 3.1.2. Knee

The flexion angle was significantly different when the participants ran at V3 wearing S1, S2 and S3 (*p* < 0.05). The extension angle was significantly different when they ran at V1 in S1, S2 and S3 (*p* < 0.05). It was significantly different when they ran at V2 in S1 and S3 (*p* < 0.05). There was a significant difference when they ran at V3 in S3 (*p* < 0.05). The flexion angle peak velocity was significantly different when they ran at V1 wearing S3 (*p* < 0.05). The extension angle peak velocity was significantly different when they ran at V2 and V3 wearing S1 and at V1 and V2 wearing S3 (*p* < 0.05).

#### 3.1.3. Ankle

The ankle dorsiflexion angle was significantly different when the participants ran at V1, V2 and V3 wearing S1, S2 and S3 (*p* < 0.05). The plantarflexion angle was significantly different when they ran at V1 wearing S1 and S2 and at V2 wearing S2 (*p* < 0.05). The dorsiflexion angle peak velocity was significantly different when they ran at V1 wearing S3 and at V2 wearing S2 (*p* < 0.05). The plantarflexion angle peak velocity was significantly different when they ran at V1 wearing S2 and at V2 wearing S2 and S3 (*p* < 0.05).

#### 3.1.4. MTP

The dorsiflexion angle was significantly different when the participants ran at V1 wearing S3, at V2 wearing S1 and S3 and at V3 wearing S1, S2 and S3 (*p* < 0.05). The plantarflexion angle was significantly different when they ran at V2 and V3 wearing S2 and S3, respectively (*p* < 0.05). The dorsiflexion angle peak velocity was significantly different when they ran at V3 wearing S2 and S3 (*p* < 0.05). The plantarflexion angle peak velocity was significantly different when they ran at V1 wearing S1 and ran at V3 wearing S3 (*p* < 0.05).

#### 3.1.5. SPM Results

The SPM results are displayed as:

(1) Hip angles were significantly different in the phases of 0–27%, 46–53% and 58–100% when the participants ran at V1 and wore S1; the phases of 11–28% and 37–55% when they ran at V1 and wore S3; the phase of 0–85% when they ran at V2 and wore S1; the phase of 0–75% when they ran at V2 and wore S2; the phase of 18–70% when they ran at V2 and wore S3; and the phase of 42–70% when they ran at V3 and wore S2. Hip angle velocities were significantly different in the phase of 35–37% when they ran at V1 and wore S1; the phases of 21–22% and 76–77% when they ran at V1 and wore S2; the phases of 35–38%, 62–65%, 66–76%, 78–81%, 83–85% and 88–90% when they ran at V1 and wore S3; the phases of 38–42%, 65–68% and 71–72% when they ran at V2 and wore S1; the phases of 90–94% and 97–100% when they ran at V2 and wore S2; the phases of 65–77% and 80–82% when they ran at V2 and wore S3; the phases of 43–47% and 58–63% when they ran at V3 and wore S1; the phase of 44–52% when they ran at V3 and wore S2; and the phase of 45–55% when they ran at V3 and wore S3. 

(2) Knee angles were significantly different in the phase of 98–100% when the participants ran at V1 and wore S3; the phase of 11–65% when they ran at V2 and wore S1; the phase of 0–3% when they ran at V2 and wore S2; and the phases of 0–8% and 88–100% when they ran at V3 and wore S1. Knee angle velocities were significantly different in the phases of 21–22% and 34–35% when they ran at V1 and wore S2; the phases of 44–45%, 58–68% and 71–73% when they ran at V1 and wore S3; the phases of 40–44% and 64–76% when they ran at V2 and wore S1; the phase of 41–49% when they ran at V3 and wore S2; and the phase of 26–31% when they ran at V3 and wore S3. 

(3) Ankle angles were significantly different in the phases of 0–77%, 45–54% and 57–100% when the participants ran at V1 and wore S1; the phase of 0–11% when they ran at V1 and wore S3; the phase of 13–44% when they ran at V2 and wore S1; the phase of 94–100% when they ran at V2 and wore S2; the phases of 1–13%, 15–17% and 22–26% when they ran at V2 and wore S3; and the phase of 5–9% when they ran at V3 and wore S1. Ankle angle velocities were significantly different in the phases of 22–25%, 27–31% and 84–85% when they ran at V1 and wore S1; the phases of 74–75% and 77–82% when they ran at V1 and wore S2; the phases of 13–14%, 52–53% and 57–65% when ran at V2 and wore S1; the phase of 88–91% when they ran at V2 and wore S2; the phase of 42–48% when they ran at V2 and wore S3; and the phase of 37–40% when they ran at V3 and wore S3.

(4) MTP angles were significantly different in the phase of 0–100% when the participants ran at V1 and wore S2; the phase of 80–94% when they ran at V1 and wore S3; the phase of 75–100% when they ran at V2 and wore S1; the phase of 0–100% when they ran at V2 and S2; the phases of 4–12% and 58–100% when they ran at V2 and wore S3; the phase of 85–95% when they ran at V3 and wore S1; the phase of 6–100% when they ran at V3 and wore S2; and the phase of 72–93% when they ran at V3 and wore S3. MTP angle velocities were significantly different in the phase of 32–41% when they ran at V1 and wore S3; the phase of 35–48% when they ran at V2 and wore S3; the phase of 0–11% when they ran at V3 and wore S1; the phase of 10–13% when they ran at V3 and wore S2; and the phase of 0–10% when they ran at V3 and wore S3.

### 3.2. The Influence of Running Velocity and LBS on SA

#### 3.2.1. Running Velocity

The knee flexion angle and flexion angle peak velocity, ankle plantarflexion angle and plantarflexion angle peak velocity, MTP dorsiflexion angle and dorsiflexion angle peak velocity were significantly different due to running velocity (*p* < 0.05). There was no interaction effect with LBS. This suggests that running velocity affected the SA of lower limb joints, regardless of the type of shoes participants wore. The statistical difference was specific as (1) the knee flexion angle was significantly different when the participants ran at V3 compared to V1 and V2 (*p* < 0.05). The knee flexion angle peak velocity was significantly different at V1, V2 and V3 (*p* < 0.05). (2) The ankle plantarflexion angle in V3 was significantly different from that in V1 and V2 (*p* < 0.05). The plantarflexion peak velocity in V1 was also significantly different from that in V3 (*p* < 0.05). (3) The MTP dorsiflexion angle was significantly different at V1, V2 and V3 (*p* < 0.05). The plantarflexion angle peak velocity was significantly different between V1 and V3 (*p* < 0.05).

#### 3.2.2. LBS

LBS had a significant effect on the MTP dorsiflexion peak velocity, and there was no interaction effect with running velocity (*p* < 0.05). This indicates that running velocity would not affect the influence of LBS on SA. MTP plantarflexion angle peak velocity was significantly different when worn in S1 and S3.

#### 3.2.3. The Interaction between Running Velocity and LBS

Interaction effects were observed in MTP. There was an interaction effect between running velocity and LBS in both the MTP plantarflexion angle and plantarflexion angle peak velocity. When ran at V2, the SA corresponding to the plantarflexion angle was significantly different between S1 and S2 shoes (*p* < 0.05). The SA of the plantarflexion angle peak velocity was significantly different between the participants ran at V1 and V2 when wearing S3 (*p* < 0.05). In addition, there was also a significant difference in the plantarflexion angle peak velocity between S2 and S3 when the participants ran at V1 (*p* < 0.05).

## 4. Discussion

This study aimed to investigate how bilateral lower limb asymmetry evolves when wearing increasing-LBS shoes at different running velocities. Asymmetry was present as evidenced by differences in angle and angle peak velocity between the hip, knee, ankle, MTP of both lower limbs and the SPM results. It also further confirmed the previous research [9,11] showing that differences exist in bilateral lower limbs. On this basis, it was vital to investigate the changes in the SA. In the exploration of the SA, we found that running velocity influenced the knee flexion angle and flexion angle peak velocity, ankle plantarflexion angle and plantarflexion angle peak velocity, MTP dorsiflexion angle and dorsiflexion angle peak velocity. Furthermore, LBS had a significant influence on the MTP dorsiflexion angle peak velocity. The interaction between running velocity and LBS existed in the MTP plantarflexion angle and plantarflexion angle peak velocity. It was found that the difference in kinematic variables of lower limb joints was not completely consistent with the change in SA. This was also reflected in the study by Jiang et al. and Gao et al. [11,12].

Studies on the influence of the running velocity and LBS on kinematic variables suggested that there were differences in the hip. However, the SA of the hip did not change significantly with running velocity and LBS. In addition, the SA of the hip was generally smaller than that of other joints, suggesting a higher symmetry. Hannah et al. pointed out that a high level of symmetry was observed for the sagittal plane of the hip [35].

The difference existed both In knee angle and angle peak velocity. In addition, the influence of running velocity on the SA of the knee mainly focused on the knee flexion angle and knee flexion angle peak velocity. In their investigation, Rodolfo et al. [36] found that there were differences in the strength of the knee flexors in the dominant and non-dominant legs. According to Ayako Higashihara et al. [37], the characteristics of the electromyographic activity of the biceps femoris and semitendinosus muscles changed as running velocity increased. Additionally, the biceps femoris activated much earlier during the stance phase than the semitendinosus. The difference between flexor and extensor muscles may be the cause of the alteration in knee kinematics, which results in the alteration in the SA with velocity. The majority of injuries sustained during running occur around or close to the knee, especially in overuse conditions, including patellar tendinitis, meniscal tears, tibial stress fractures and patellofemoral pain syndrome [38]. The knee kinematic difference is frequently used by medical professionals, physiotherapists or strength and conditioning specialists to quantify the functional deficit caused by a knee injury and/or surgery, like an anterior cruciate ligament injury, to track the effectiveness of sports rehabilitation programs and establish baseline data for readiness to return to play after an injury [39].

The influence of running velocity on the SA of the ankle was primarily focused on the plantarflexion angle and plantarflexion angle peak velocity. As the largest articular joint of the distal lower limb, differences in side-to-side force production may influence joint kinematics, potentially increasing the injury risk [40]. Additionally, the changes in asymmetries might be seen as a compensatory mechanism to keep the gait stable when running [11]. As a consequence, changes in the difference and SA of the ankle plantarflexion angle and plantarflexion angle peak velocity may serve as a mechanism of self-protection during running. When the participants ran at V1, the SA corresponding to the ankle plantarflexion angle and plantarflexion angle peak velocity were generally smaller than when the participants ran at V2 and V3. This may imply that slower velocities are preferable for recreational runners.

Running velocity influenced the SA of the MTP dorsiflexion angle and dorsiflexion angle peak velocity significantly. Additionally, the SA generally increased with the increase in running velocity. Limb dominance is believed to be task-dependent. It has been proposed that the functions of the lower limbs (such as those seen in muscle activity and joint kinematics) lead to a local asymmetry [41]. Asymmetry in the plantar pressure of the MTP as well as functional differences between the dominant and non-dominant legs may be to blame for changes in the SA of the MTP dorsiflexion angle and dorsiflexion angle peak velocity. In the study of plantar pressure symmetry by Gao et al., it was found that the metatarsal pressure and the corresponding SA increased with increasing velocity [12]. Furthermore, greater dorsiflexion was one of the factors contributing to elevated metatarsal pressure [42]. Changes in the MTP dorsiflexion angle and dorsiflexion angle peak velocity may be related to changes in the SA due to metatarsal pressure. MTP serves as the foundation of support once the heel has lifted while running [43]. Moving at a higher speed requires the MTP to create significantly greater propulsion [44]. Therefore, the MTP is significantly affected by running velocity. The asymmetry of the MTP may be the underlying cause of metatarsal stress fractures [45]. Additionally, we discovered that, typically speaking, the SA rose as running velocity rose. This might imply that casual runners are more suited to running at a slower pace. Additionally, the MTP dorsiflexion angle peak velocity was mostly where LBS had an effect on the SA. Furthermore, the SA increased with the increase in LBS. Willwacher et al. [46] demonstrated that increasing the longitudinal bending stiffness in running shoes led to a significant anterior shift of all lower limb lever arms, and this effect was greater at the more distal joints. This could be the cause of LBS’s effects in this investigation, which were limited to the MTP’s SA. Gait asymmetry in healthy individuals has been referred to as functional asymmetry, which describes limb asymmetry based on the distinction of fundamental functions, namely stabilization or propulsion [20]. Differences in muscle function and strength could be the source of the asymmetry of the bilateral MTP. Interestingly, there were interaction effects between LBS and running velocity on the MTP plantarflexion angle and plantarflexion angle peak velocity. The result for the MTP plantarflexion angle showed that when the participants ran at V2, the SA corresponding to S2 was significantly smaller than that corresponding to S1. In addition, the result for MTP plantarflexion angle peak velocity showed that the SA corresponding to S2 was significantly smaller than that corresponding to S3 when the participants ran at V1. When they ran in S3, the SA corresponding to V2 was significantly smaller than that corresponding to V1. In their study, Cornelis et al. [47] found that recreational runners performed better when they ran at velocities between 10 and 13 km/h, which is broadly comparable to the improved symmetry in our results for running at low and moderate velocities. Increased LBS can protect the MTP by limiting the plantarflexion of the MTP. However, larger LBS required greater muscle strength to overcome the stiff carbon-fiber plate [8]. When the threshold is exceeded, running performance may deteriorate [48]. This suggests that there may be an optimal LBS. In addition, the results of this study show that the influence of LBS on the SA was nearly U-shaped when run at low or moderate velocity. This further indicates that there was an optimal LBS for the influence of LBS on the SA of the MTP. This could mean that recreational runners would benefit more from using shoes with modest LBS when running at low-to-moderate velocities since they would have better symmetry and a better chance of avoiding sports injuries.

Although our study is very novel, there are some limitations. Only kinematic data of lower extremity joints were discussed in this study. The asymmetry of the dynamic variables of the lower limb joints was not discussed. The following research can explore the asymmetry of lower limb dynamics variables and expand the sample to include novice runners and competitive runners.

## 5. Conclusions

These findings indicate the existence of bilateral lower limb asymmetry. Meanwhile, it has been established that running velocity and LBS affect the asymmetry of the lower limb. Additionally, there was an interaction between running velocity and LBS on the asymmetry of lower limb. These findings can provide some information for sports injuries, such as metatarsal stress fractures and anterior cruciate ligament injuries. They can also provide some useful suggestions for running velocities and running shoes.

## Figures and Tables

**Table 1:** The information of shoes.

	S1	S2	S3
LBS value (Nm/rad)	2.7	5.0	8.6
Appearance	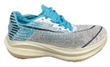	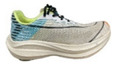	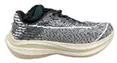

**Table 2:** The influence of running velocity and LBS on lower limb joints’ kinematic variables.

Joint Kinematics			S1		*p*-Value	S2		*p*-Value	S3		*p*-Value
			L	R		L	R		L	R	
Hip	Extension angle (◦)	V1	49.07 ± 10.11	44.01 ± 8.92	**0.033**	48.27 ± 9.11	41.90 ± 7.83	**0.010**	47.56 ± 9.83	40.28 ± 8.50	**<0.001**
		V2	48.71 ± 14.84	46.05 ± 10.95	0.094	53.79 ± 2.46	50.15 ± 8.82	0.213	53.84 ± 10.39	50.80 ± 9.41	**0.037**
		V3	66.87 ± 7.29	61.32 ± 10.41	0.142	67.94 ± 7.58	66.06 ± 9.41	0.451	63.99 ± 8.34	67.77 ± 7.39	0.287
	Extension angle velocity (rad/s)	V1	6.47 ± 0.44	6.16 ± 0.97	0.433	5.97 ± 0.23	6.18 ± 0.46	0.130	6.05 ± 0.33	5.94 ± 0.49	0.595
		V2	6.99 ± 0.66	6.99 ± 0.56	0.977	7.12 ± 0.60	6.99 ± 0.48	0.699	7.61 ± 0.48	6.90 ± 0.65	**0.004**
		V3	9.75 ± 0.88	9.37 ± 0.97	0.519	9.49 ± 0.60	9.42 ± 0.50	0.818	9.13 ± 0.93	9.45 ± 0.52	0.434
Knee	Flexion angle (◦)	V1	29.10 ± 4.19	27.36 ± 4.34	0.135	26.98 ± 2.45	27.75 ± 2.67	0.215	28.77 ± 3.97	28.06 ± 4.85	0.689
		V2	26.20 ± 3.03	26.37 ± 4.63	0.864	25.22 ± 3.52	25.95 ± 1.41	0.573	26.49 ± 6.02	25.49 ± 2.44	0.653
		V3	18.13 ± 2.57	15.58 ± 4.22	**0.012**	19.01 ± 2.07	15.01 ± 2.35	**0.003**	21.38 ± 3.05	15.99 ± 2.86	**0.007**
	Extension angle (◦)	V1	25.06 ± 8.81	19.03 ± 9.29	**0.005**	22.16 ± 7.37	19.23 ± 5.52	**0.034**	23.04 ± 7.62	15.91 ± 8.35	**<0.001**
		V2	22.56 ± 11.36	17.80 ± 10.78	**0.002**	20.16 ± 4.55	18.28 ± 9.88	0.488	20.70 ± 12.14	17.66 ± 11.39	**0.014**
		V3	22.22 ± 10.78	16.42 ± 10.89	**0.007**	23.97 ± 10.73	22.70 ± 10.66	0.632	21.21 ± 9.76	25.48 ± 6.60	0.192
	Flexion angle velocity (rad/s)	V1	4.99 ± 1.06	4.12 ± 1.47	0.058	4.28 ± 1.14	4.32 ± 1.28	0.938	4.51 ± 1.11	3.37 ± 1.28	**0.003**
		V2	9.39 ± 1.67	8.30 ± 1.83	0.063	8.93 ± 1.66	8.31 ± 0.58	0.276	9.58 ± 2.72	8.02 ± 0.56	0.122
		V3	9.30 ± 2.79	8.38 ± 2.54	0.482	9.72 ± 2.41	8.13 ± 1.90	0.236	10.73 ± 2.18	8.12 ± 2.18	0.065
	Extension angle velocity (rad/s)	V1	4.99 ± 1.06	4.12 ± 1.47	0.058	4.28 ± 1.14	4.32 ± 1.28	0.938	4.51 ± 1.11	3.37 ± 1.28	**0.003**
		V2	4.67 ± 1.91	3.91 ± 1.45	**0.008**	4.33 ± 0.88	3.86 ± 1.10	0.205	4.53 ± 2.06	3.62 ± 1.47	**0.002**
		V3	5.38 ± 2.09	4.28 ± 1.87	**0.027**	5.98 ± 2.20	5.13 ± 1.56	0.251	5.32 ± 2.01	5.56 ± 0.99	0.700
Ankle	Dorsiflexion angle (◦)	V1	24.30 ± 2.10	18.25 ± 1.57	**<0.001**	22.34 ± 1.89	18.53 ± 0.98	**0.001**	23.87 ± 1.71	18.78 ± 0.92	**<0.001**
		V2	19.61 ± 2.23	17.28 ± 1.66	**0.004**	22.09 ± 3.41	17.66 ± 1.52	**0.017**	23.99 ± 2.21	19.74 ± 1.16	**0.001**
		V3	21.43 ± 3.53	17.34 ± 1.72	**0.011**	23.04 ± 3.10	19.34 ± 1.83	**0.030**	22.54 ± 2.35	19.14 ± 1.62	**0.004**
	Plantarflexion angle (◦)	V1	42.45 ± 4.62	39.90 ± 4.01	**0.048**	43.59 ± 4.42	38.43 ± 4.79	**0.005**	41.11 ± 5.91	38.12 ± 4.97	0.096
		V2	40.01 ± 8.02	39.89 ± 5.68	0.967	46.45 ± 1.60	40.94 ± 6.34	**0.047**	44.88 ± 5.35	44.01 ± 4.92	0.491
		V3	43.53 ± 8.80	34.87 ± 6.39	0.073	47.37 ± 7.95	45.66 ± 9.13	0.714	46.00 ± 4.77	43.67 ± 7.91	0.552
	Dorsiflexion angle velocity (rad/s)	V1	6.12 ± 0.71	5.71 ± 0.79	0.323	7.37 ± 2.13	7.24 ± 2.05	0.764	8.23 ± 2.57	7.10 ± 1.87	**0.038**
		V2	6.14 ± 0.72	6.07 ± 1.43	0.897	8.50 ± 2.71	6.69 ± 1.34	**0.048**	8.67 ± 2.94	8.28 ± 2.79	0.616
		V3	8.32 ± 2.99	7.31 ± 1.59	0.343	9.75 ± 3.68	8.80 ± 2.03	0.293	10.19 ± 3.65	9.26 ± 3.33	0.530
	Plantarflexion angle velocity (rad/s)	V1	8.52 ± 0.52	7.80 ± 0.66	0.127	8.69 ± 0.65	7.38 ± 0.51	**0.003**	8.14 ± 0.80	7.45 ± 0.84	0.138
		V2	10.07 ± 1.53	9.15 ± 0.70	0.134	11.26 ± 1.10	8.77 ± 0.62	**0.001**	11.41 ± 1.13	9.65 ± 0.46	**<0.001**
		V3	13.20 ± 2.64	11.49 ± 2.16	0.172	14.73 ± 2.37	13.55 ± 1.82	0.321	15.29 ± 3.24	12.73 ± 1.97	0.124
MTP	Dorsiflexion angle (◦)	V1	15.03 ± 3.82	15.90 ± 3.89	0.614	14.91 ± 2.05	17.20 ± 4.22	0.059	11.56 ± 1.67	13.40 ± 1.33	**0.046**
		V2	13.63 ± 4.15	20.11 ± 1.88	**<0.001**	18.10 ± 3.30	19.45 ± 2.18	0.420	13.10 ± 0.86	18.60 ± 2.55	**<0.001**
		V3	16.95 ± 3.68	25.28 ± 2.29	**<0.001**	15.85 ± 6.10	24.57 ± 4.20	**0.020**	13.67 ± 4.22	22.17 ± 1.89	**<0.001**
	Plantarflexion angle (◦)	V1	14.05 ± 4.09	15.65 ± 4.42	0.534	13.29 ± 3.37	12.60 ± 3.47	0.621	8.86 ± 3.67	8.88 ± 2.34	0.988
		V2	14.84 ± 5.37	13.08 ± 6.00	0.608	12.82 ± 2.53	15.30 ± 2.76	**0.011**	9.89 ± 1.84	12.72 ± 2.70	**0.050**
		V3	17.71 ± 5.40	19.89 ± 7.04	0.454	13.53 ± 7.40	20.37 ± 3.02	**0.013**	9.77 ± 1.64	14.83 ± 5.49	**0.020**
	Dorsiflexion angle velocity (rad/s)	V1	3.53 ± 0.72	3.53 ± 0.49	0.985	2.81 ± 0.73	3.78 ± 2.46	0.205	2.83 ± 0.68	3.35 ± 0.63	0.214
		V2	3.53 ± 0.72	3.53 ± 0.49	0.985	2.81 ± 0.73	3.78 ± 2.46	0.205	2.83 ± 0.68	3.35 ± 0.63	0.214
		V3	5.18 ± 2.08	6.05 ± 1.03	0.226	5.33 ± 1.90	7.99 ± 1.55	**0.004**	3.82 ± 1.16	6.96 ± 2.53	**0.001**
	Plantarflexion angle velocity (rad/s)	V1	6.66 ± 1.80	8.44 ± 1.58	**0.039**	8.00 ± 2.76	7.13 ± 1.68	0.360	4.84 ± 2.61	4.23 ± 1.32	0.618
		V2	7.57 ± 1.01	8.14 ± 3.59	0.662	9.50 ± 2.42	8.83 ± 1.99	0.587	6.59 ± 0.99	7.65 ± 2.05	0.155
		V3	12.84 ± 4.62	15.06 ± 3.57	0.236	11.31 ± 4.79	15.73 ± 3.14	0.064	8.29 ± 1.64	11.16 ± 2.82	**0.022**

L = left lower limb, R = right lower limb. Data in bold in the table indicate statistical significance, *p* < 0.05. Value: mean ± SD.

**Table 3:** The influence of running velocity and LBS on SA.

Joint Kinematics			Symmetry Angle [%]			V			S			V·S		
			S1	S2	S3	*p*	*F*	*η* ^2^	*p*	*F*	*η* ^2^	*p*	*F*	*η* ^2^
Hip	Extension	V1	4.92 ± 2.75	5.22 ± 3.85	5.27 ± 2.40	0.077	2.650	0.061	0.920	0.084	0.002	0.215	1.480	0.068
		V2	2.49 ± 1.40	4.90 ± 3.35	2.58 ± 1.91									
		V3	4.93 ± 4.16	3.03 ± 1.94	4.42 ± 3.18									
	Extension angle velocity	V1	5.35 ± 3.44	1.95 ± 1.06	2.50 ± 2.02	0.702	0.355	0.009	0.119	2.189	0.051	0.078	2.181	0.097
		V2	2.04 ± 1.77	3.09 ± 2.55	3.32 ± 2.32									
		V3	4.36 ± 3.91	2.32 ± 2.43	3.43 ± 2.54									
Knee	Flexion	V1	3.27 ± 2.42 ^c^	1.91 ± 0.99 ^c^	4.95 ± 2.74 ^c^	**0.001**	7.753	0.161	0.067	2.798	0.065	0.852	0.337	0.016
		V2	3.25 ± 1.98 ^c^	4.02 ± 2.61 ^c^	6.48 ± 3.54 ^c^									
		V3	6.31 ± 6.74 ^ab^	8.14 ± 5.19 ^ab^	9.26 ± 7.91 ^ab^									
	Extension	V1	12.01 ± 9.34	6.69 ± 3.31	13.80 ± 9.10	0.572	0.562	0.014	0.892	0.114	0.003	0.449	0.934	0.044
		V2	9.54 ± 8.93	12.58 ± 10.83	7.48 ± 6.75									
		V3	12.93 ± 1.18	11.88 ± 11.75	13.05 ± 12.54									
	Flexion angle velocity	V1	25.18 ± 2.67 ^bc^	28.23 ± 2.11 ^bc^	26.40 ± 2.92 ^bc^	**<0.001**	72.845	0.643	0.978	0.022	0.001	0.652	0.617	0.030
		V2	5.74 ± 5.20 ^ac^	4.21 ± 3.73 ^ac^	6.66 ± 3.03 ^ac^									
		V3	12.73 ± 10.76 ^ab^	12.04 ± 7.63 ^ab^	10.86 ± 10.55 ^ab^									
	Extension angle velocity	V1	8.85 ± 8.77	7.26 ± 6.07	11.59 ± 8.01	0.078	2.631	0.061	0.888	0.119	0.003	0.694	0.558	0.027
		V2	7.00 ± 3.57	7.36 ± 7.57	7.47 ± 5.92									
		V3	10.43 ± 5.95	13.26 ± 9.87	10.91 ± 9.14									
Ankle	Dorsiflexion	V1	9.07 ± 3.99	6.02 ± 3.02	7.49 ± 3.07	0.370	1.008	0.024	0.731	0.314	0.008	0.142	1.772	0.080
		V2	4.39 ± 2.44	7.71 ± 5.63	6.05 ± 3.64									
		V3	7.73 ± 4.10	6.39 ± 5.15	5.07 ± 3.83									
	Plantarflexion	V1	2.58 ± 1.87 ^c^	4.35 ± 3.16 ^c^	4.24 ± 2.44 ^c^	**0.001**	7.755	0.161	0.130	2.095	0.049	0.184	1.594	0.073
		V2	5.24 ± 6.58 ^c^	5.14 ± 4.71 ^c^	1.95 ± 2.08 ^c^									
		V3	11.58 ± 5.95 ^ab^	8.20 ± 5.96 ^ab^	5.47 ± 7.22 ^ab^									
	Dorsiflexion angle velocity	V1	5.45 ± 3.48	3.54 ± 2.92	5.61 ± 3.93	0.153	1.920	0.045	0.325	1.141	0.027	0.459	0.915	0.043
		V2	6.79 ± 4.32	7.37 ± 7.11	5.85 ± 3.90									
		V3	10.70 ± 5.97	6.80 ± 4.49	5.47 ± 8.78									
	Plantarflexion angle velocity	V1	3.62 ± 2.11 ^c^	5.32 ± 3.40 ^c^	4.92 ± 2.77 ^c^	**0.043**	3.279	0.075	0.670	0.403	0.010	0.625	0.655	0.031
		V2	5.34 ± 3.71	7.73 ± 4.30	5.17 ± 2.19									
		V3	9.00 ± 6.28 ^a^	7.31 ± 4.56 ^a^	7.06 ± 8.08 ^a^									
MTP	Dorsiflexion	V1	7.32 ± 7.34 ^bc^	4.25 ± 4.20 ^bc^	6.10 ± 4.67 ^bc^	**<0.001**	16.748	0.293	0.626	0.472	0.012	0.123	1.872	0.085
		V2	13.09 ± 7.61 ^ac^	6.94 ± 4.32 ^ac^	10.65 ± 3.98 ^ac^									
		V3	13.00 ± 5.83 ^ab^	17.67 ± 7.83 ^ab^	15.84 ± 7.42 ^ab^									
	Plantarflexion	V1	11.41 ± 8.89	7.50 ± 5.08	14.78 ± 9.21	0.303	1.211	0.029	0.217	1.556	0.037	**0.033**	2.757	0.120
		V2	19.47 ± 14.11 ^f^	7.10 ± 3.35 ^f^	9.57 ± 6.91									
		V3	12.65 ± 7.74	16.57 ± 11.03	15.09 ± 7.67									
	Dorsiflexion angle velocity	V1	7.48 ± 6.07 ^ce^	9.36 ± 8.60 ^c^	9.85 ± 7.54 ^cd^	**0.019**	4.164	0.093	**0.014**	4.516	0.100	0.460	0.913	0.043
		V2	7.00 ± 5.03 ^e^	6.35 ± 6.56	15.50 ± 5.77 ^d^									
		V3	11.11 ± 8.16 ^ae^	14.04 ± 8.92 ^a^	17.40 ± 7.99 ^ad^									
	Plantarflexion angle velocity	V1	9.92 ± 5.76	8.30 ± 7.78 ^g^	18.77 ± 12.51 ^gh^	0.690	0.373	0.009	0.787	0.240	0.006	**0.038**	2.662	0.116
		V2	14.69 ± 12.95	10.44 ± 6.36	8.05 ± 5.42 ^h^									
		V3	11.35 ± 9.90	15.58 ± 8.05	12.40 ± 6.88									

V = running velocity, S = shoes, V·S = the interaction between running velocity and shoes. ^a^ = significantly different with V1, ^b^ = significantly different with V2, ^c^ = significantly different with V3, ^d^ = significantly different with S1, ^e^ = significantly different with S3, ^f^ = have a significant difference between V2/S1 and V2/S2, ^g^ = a significant difference between V1/S2 and V1/S3, ^h^ = a significant difference between S3/V1 and S3/V2. Data in bold in the table indicate statistical significance, *p* < 0.05. Value: mean ± SD.

## Data Availability

The data that support the findings of this study are available on reasonable request from the corresponding author. The data are not publicly available due to privacy or ethical restrictions.
